# Heterogeneous impact of anti-bullying laws on school bullying in the State of Georgia

**DOI:** 10.1371/journal.pone.0332028

**Published:** 2025-09-26

**Authors:** Mona Ahmadiani, Pourya Valizadeh, Genti Kostandini, Jeffrey L. Jordan

**Affiliations:** 1 Department of Agricultural Economics, Texas A&M University, College Station, Texas, United States of America; 2 Department of Agricultural and Applied Economics, University of Georgia, Athens, Georgia, United States of America; University of Study of Bari Aldo Moro, ITALY

## Abstract

The increasing prevalence of bullying behavior has made anti-bullying laws (ABL) and policies a public health priority in the United States. Using 2007–2016 data from the Georgia Student Health Survey 2.0 on 6–12 grade public school students, we examine the relationship between two ABLs—with different levels of stringency—and the prevalence of bullying behavior in the state of Georgia. We find that although the implementation of both Georgia’s ABLs is associated with significant declines in the prevalence of bullying victimization, the more stringent ABL is associated with substantially larger reductions in reported cases. Our heterogeneity analysis based on external environment characteristics shows that the more stringent ABL is associated with smaller reductions in bullying victimization incidents in schools with a higher share of students enrolled in the federally subsidized school lunch program. The more stringent ABL is also associated with larger increases in bystanders’ willingness to intervene in school districts with higher poverty rates among children aged 5–17. Lastly, we analyze disparities in bullying victimization reporting at the school level and find that students tend to report higher bullying victimization rates than school personnel, and that the two ABLs are associated with larger reductions in the student reporting than in personnel reporting. Our results also show that the reporting disparities fade away when ABLs are in place, providing further evidence of the role of ABLs in mitigating bullying victimization reporting discrepancies.

## Introduction

Youth bullying—defined as the repeated intentional harm caused by peers that involves a real or perceived imbalance of power between the perpetrator and the victim—is a major public health concern in the United States (US) [1–3]. Youth bullying, which often occurs in a school setting (school bullying) takes several forms, from verbal intimidation and cyberbullying to physical aggression, and has its roots in a number of psychological, behavioral, emotional, and physical adjustment problems [4,5]. As a form of negative social interaction, bullying is also found to increase the risk of adverse mental health outcomes (e.g., depression, suicidal ideation, muscle dysmorphic disorder) [6–9], impair physical development [10], lower educational attainment and earnings [11–13], and adversely affect bullying victims’ psychological development [14–23].

The prevalence of school-based bullying victimization in the US has been declining from an estimated 28.5% in 2005 to 20% in 2017 primarily due to extensive anti-bullying laws (ABLs) introduced at the federal and state levels and increased awareness of the bullying problem [24]. However, the introduction of electronic communication into classrooms has opened doors for cyberbullying, which has substantially expanded the incidence of bullying [25]. Musu-Gillette et al. (2018) [26] note that 20% of students aged 12–18 report bullying victimization at school, over half of whom are cyberbullying victims. Based on the 2016–17 report by the US National Center for Education Statistics (NCES) [27], which examines the percentage of cyberbullying among all bullying incidents reported by students aged 12 to 18, 21% of females compared to 7% of males, 17% of White students compared to 12% of students of other races, and 19% of high school students compared to 12% of middle school students reported being bullied online or by text.

The emerging issue of cyberbullying has led to the passage of cyberbullying laws across US states. Although several studies have investigated the impact of US ABLs [28–32], the existing literature remains inconclusive regarding the impact of ABLs in general or cyberbullying laws in particular on bullying behavior and bullying reporting. For instance, using Youth Risk Behavior Surveys, Dasgupta (2019) [31] investigates the change in physical violence among high school students as a proxy for cyberbullying across US states from 2001 to 2013 and finds a positive association between cyberbullying laws and bullying victimization reporting in the range of 11%–17%. In contrast, using the same data, Sabia and Bass (2017) [30] find that state ABLs are associated with an 8% to 12% reduction in bullying victimization.

These divergent findings stem in part from differences in empirical approaches. Dasgupta (2019) [31] focuses on effective dates of ABLs across US states, thereby giving equal weight to different policies across states regarding their stringency (e.g., weak, moderate, and strong policies), and interprets the estimated positive relationship with an emphasis on the main objective of ABLs, which is to promote higher bullying victimization reporting. Sabia and Bass (2017) [30], on the other hand, exploit the within-state policy variation in terms of the stringency of ABLs to comprehensively examine their impact on school safety, bullying victimization, and youth violence. Nikolaou (2017) [32] investigates the effectiveness of various types of ABLs at the school level from 2000 to 2015 and finds that schools in states with such laws had up to 8.4% fewer reported school bullying victimization incidents compared to schools in states without ABLs. Other studies find virtually no relationship between ABLs and violence and victimization of children in a school setting [28,29].

In this paper, we focus on the ABLs in the state of Georgia and comprehensively examine their relationship with bullying behavior among 6th–12th grade public school students. Georgia was the first US state to enact an ABL in 1999 in the aftermath of the Columbine High School shooting and in response to bullying-related suicides. In August 2010, the Georgia General Assembly updated its original ABL to a more comprehensive and stricter version that required the Georgia Department of Education (GaDOE) to develop a model bullying policy. In terms of stringency, this enacted law, which became effective in 2011, expanded the definition of bullying to include different cases of intimidation and harassment, including the act of cyberbullying. The 2010 law was also stricter compared to the status quo, as not only did it prohibit bullying for all schools serving grades six through 12, but also required age-appropriate consequences and interventions for all schools. According to GaDOE, “The law requires that through appropriate due process, disciplinary hearing officers, panels, or tribunals assign students in grades six through 12 to alternative schools when such students have committed an offense of bullying for the third time in a school year."

In 2015, “The End to Cyberbullying Act” was adopted by the state through House Bill 131 (HB131) and provides that any act of cyberbullying would be considered, whether or not such an electronic act originated on school property or with school equipment (GaDOE 2015). Given that electronic devices and technology have introduced new forms of bullying, Georgia law classifies cyberbullying as an act of bullying “transmitted in whole or in part by a wire, radio, electromagnetic, photo electronic, or photo-optical system." This enactment marked a notable increase in legal stringency, as prior to 2015, most cases of cyberbullying could not face disciplinary action while they were taking place outside school property.

We examine three different angles of the impact of Georgia’s ABLs on students’ bullying behavior. First, we explore whether and how Georgia’s more recent ABLs with stronger degrees of *stringency* (i.e., the 2010 and 2015 ABLs) were associated with significant changes in the prevalence of bullying behavior among 6th–12th grade public school students. In our analysis, we consider the period before 2011 as the *status quo*, as we are unable to investigate the impact of the 1999 ABL due to limited data availability. Specifically, we examine the impact on three parties involved in a bullying incident, including students experiencing bullying victimization, students engaging in bullying perpetration, and students indicating willingness to intervene as bystanders. Although bullying is often considered to primarily affect the direct victims, perpetrators and bystanders also suffer substantially from such behavior [33–35]. While victims are more prone to youth violence and nonfatal injuries [36,37], perpetrators suffer from a higher risk of suicidal ideation [38]. Moreover, bullying prevention and intervention studies emphasize the role of bystanders and show that students’ willingness to intervene depends on their perception of the level of harm as well as their sociodemographic and psychological characteristics [33,35].

Second, we examine heterogeneity in the relationship between Georgia’s ABLs and the prevalence of bullying outcomes with respect to disadvantaged external environments. Exposure to chronic stress that includes living in impoverished neighborhoods, is hypothesized to be correlated with youth violence [37]. This hypothesis has been tested empirically in previous studies by looking at location determinants of bullying outcomes, such as the level of urbanization and community safety, but no significant relationship has been found [39]. In this study, we revisit the possibility of a relationship between a disadvantaged external environment and bullying outcomes by utilizing the hierarchical format of our unique bullying data obtained from the Georgia School Health Survey 2.0 (GSHSII), which includes data on nearly 95% of students aged 13 to 17 who were enrolled in Georgia’s public schools.

Third, we contribute to the literature by exploring the presence of disparities in bullying victimization reporting between students and school personnel at the school level, as well as the potential impact of Georgia’s ABLs in eliminating this disparity. We leverage our student-level bullying data and couple it with school personnel’s reported incidents of bullying-related disciplinary actions to address an important limitation of the literature regarding the differences between students’ experience and school personnel’s reporting of bullying victimization. While previous studies [32,40] have used information on school officials (principals), our study is the first that simultaneously analyzes bullying victimization reporting from two sources of students and school personnel.

Although previous national-level studies provide suggestive empirical evidence on the effectiveness of state policy interventions on bullying victimization [30–32], they fall short of exploring potential heterogeneity of this impact at the school, school district, and county levels. Utilizing data from GSHSII, we investigate the heterogeneity in the relationship between ABLs and the prevalence of bullying behavior with respect to confounding variables at the school district and county levels. We combine GSHSII with other data from GaDOE, such as school financial reports, students’ enrollment by race/ethnicity, the percentage of students participating in the free and reduced-price school lunch program, and school personnel’s reported incidents of bullying-related disciplinary actions. We also utilize school district- and county-level determinants of a disadvantaged external environment, including poverty from the Small Area Income and Poverty Estimates Program (SAIPE) of the US Census Bureau, and violent crime rates from the FBI Uniform Crime Rate Report.

We find that while both the 2010 and 2015 ABLs were associated with significant reductions in bullying victimization, the implementation of the more stringent ABL of 2015 was associated with a larger decrease of about 10–20 percentage points, depending on the model used. In terms of the differential relationship between ABLs and bullying outcomes by the external environment characteristics, we find that, while the more stringent ABL of 2015 is associated with smaller reductions in bullying victimization incidents in schools with a higher share of students enrolled in the federally subsidized school lunch program, it is associated with larger increases in bystanders’ willingness to intervene in school districts with higher poverty rates among children aged 5–17. Our school-level analysis of disparities in bullying reporting finds that students report higher bullying victimization rates than school personnel, and that the two ABLs are associated with larger reductions in student reporting than in school personnel reporting. Our results further indicate that the reporting disparities fade away following the implementation of the 2010 and 2015 ABLs, providing further evidence of the effectiveness of ABLs in mitigating bullying reporting discrepancies.

## Data

We obtain student-level bullying behavior data from the GSHSII, administered by GaDOE, for the period starting with the school year 2007 (i.e., August 2007–May 2008) and ending in the school year 2016 (i.e., August 2016–May 2017). We have obtained the deidentified data from GaDOE in two separate requests, one in 2017 and one in 2018. The GSHSII is a self-administered survey that provides relevant and useful information on the quality and characteristics of school life (e.g., safety and health issues) to schools and public health professionals to develop and implement prevention and intervention strategies that may affect students’ achievement. This data includes over 4.7 million student-by-school year observations, corresponding to nearly 95% of 6th–12th grade students, attending 356 public schools, within 185 school districts in Georgia. In the GSHSII, students are not linked by unique identifiers across school years. Thus, we are not able to identify unique students and track them across school years. We merge GSHSII with school and external environment characteristics data—available at the school district and county level—from GaDOE and other data sources, creating a hierarchical data structure. Below, we discuss our data in further detail and describe the construction of variables used in the empirical analysis.

### Construction of outcome variables

The GSHSII contains a set of questions regarding whether 6th–12th grade students are involved in school bullying as victims or bullies or would be willing to get involved as bystanders to help victims. We use this data to create three different outcome variables for bullying victimization, perpetration, and bystander intervention. The bullying questions in surveys corresponding to the school years (SYs) 2007–2013 collect information about the number of days the student was being bullied: “During the past 30 days, I have been bullied or threatened by other students . . . days,” and students can choose from 0 to 30 days of being bullied. In the SY 2014 survey, students were asked “In the last 30 days, I have been bullied or threatened by other students,” and could answer on a range from “strongly disagree” to “strongly agree.” In SYs 2015 and 2016 surveys, students were asked the same question but were given five different response categories of “never,” “once or twice,” “a few times,” “many times,” and “every day.” We harmonize these three versions of the question to create a bullying victimization dummy variable, coded as 1 if a student is 1) experiencing more than 0 days of bullying for the SYs 2007–2013 surveys, 2) answering “strongly agree” or “agree” in the SY 2014 survey, and 3) answering “once or twice,” “a few times,” “many times,” or “every day” in SYs 2015 and 2016 surveys, and zero otherwise.

Similarly, using the bullying bystander question in SYs 2007–2016 surveys, asking students if they “would help someone who is being bullied," we generate a bystander intervention dummy variable taking on a value of 1 if students responded “strongly agree" or “somewhat agree" and a value of 0 if they responded “somewhat disagree" or “strongly disagree." Moreover, considering the direct impact of ABLs on bullying perpetration through school disciplinary actions, we also constructed a bullying perpetration dummy variable based on the question in the SYs 2007–2013 surveys asking students about the number of days they had bullied or threatened other students within the past 30 days. We note that GSHSII for SYs 2014–2016 did not include a bullying perpetration question. Thus, our analysis of the impact of Georgia’s ABLs on the prevalence of bullying perpetration is limited to the SYs 2007–2013 period, encompassing the 2010 ABL.

We also construct categorical variables to capture the intensity of bullying behavior for victims, perpetrators, and bystanders in four categories. First, to create the categorical bullying victimization variable for SYs 2007–2013, we use 0 days of being bullied as the first category of the respective categorical variables, 1 to 2 days as the second category, 3 to 5 days as the third category, and more than 5 days as the fourth category; for SY 2014, we use categories of “strongly disagree” to “strongly agree” as the four categories; and for SYs 2015 and 2016, the categories represent never being bullied (category 1), being bullied a few times (category 2), many times (category 3), and every day (category 4), respectively. Similarly, to construct the categorical variable for bullying perpetration for SYs 2007–2013, we use 0 days of bullying other students as the first category, 1 to 2 days as the second category, 3 to 5 days as the third category, and more than 5 days as the fourth category. Finally, for the bullying bystander, categories include self-descriptive scales of “strongly disagree” (category 1) to “strongly agree” (category 4).

### Construction of policy variables

Georgia public schools administer the GSHSII and are required to comply with the state ABLs. Therefore, we can use the variation in bullying behavior among public school students to investigate the relationship between the 2010 and 2015 ABLs and the bullying outcome variables defined above. Because the original ABL introduced in the state of Georgia was enacted in 1999, in our analysis, the survey years covering SYs 2007 (ending in May 2008) through 2010 (ending in May 2011) correspond to the *status quo*, when the 1999 ABL was already in place. We construct two indicator variables, denoted by *ABL*^2010^ and *ABL*^2015^, that reflect the two consecutive ABLs of 2010 and 2015, respectively. The 2010 ABL became effective in August 2011. Thus, the *ABL*^2010^ equals one for SY 2011 (starting in August 2011) through SY 2016. Likewise, because the 2015 ABL, with a more stringent policy toward reducing cyberbullying, became effective in August 2015, the *ABL*^2015^ equals one for SYs 2015 and 2016, and zero otherwise.

### Individual, school, and external environment characteristics

We combine GSHSII, which contains student-level sociodemographic and school climate variables, with school characteristic data (collected from GaDOE), including school financial reports that provide pupil service expenditures at the school level (an indicator of the availability of resources to address bullying-related issues), student enrollment reports by race/ethnicity that we use to analyze the impact of being in a minority racial/ethnic group by defining a “racial minority” variable (equaling 1 when a student has a racial orientation other than the school majority), the number of disciplinary incidents as a result of bullying or intimidation (an indicator of actual bullying behavior reported to school officials), and the percentage of recipients of free and reduced-price school lunches as an indicator of poverty at the school level. As a result, we expect a steady relationship between the number of bullying incidents reported by students and the number of bullying or intimidation disciplinary actions reported by school officials to GaDOE.

Finally, we merge the student-level bullying data with school district-level determinants of a disadvantaged external environment, including poverty rates from the Small Area Income and Poverty Estimates Program (SAIPE) of the US Census Bureau and the indicators of rural, suburban, or urban regions (city or towns) obtained from the NCES. We also merge in county-level violent crime rates, using a school district–county crosswalk we constructed, to capture broader environmental risk factors that may influence bullying behavior.

### Descriptive statistics

Our final analysis sample includes over 3.53 million student-by-school-year observations with no missing covariate information over the SYs 2007–2016. Table 1 presents summary statistics for the bullying outcome variables used in this study for the full sample and the three periods of interest corresponding to the *status quo* (i.e., SYs 2007–2010) and the passage of the 2010 and 2015 ABLs (i.e., SYs 2011–2016 and 2015–2016, respectively). In the full sample, about 20% of Georgia’s public school students reported being bullied at least once within the past 30 days in a given school year, and about 7% of bullying victims stated being bullied every day (i.e., category 4). On the other hand, about 8% of students reported that they had bullied other students at least once during the past 30 days; and among these students, 12% reported bullying other students every day (i.e., category 4). Lastly, approximately 89% of students indicated a willingness to intervene as a bystander to help someone who is being bullied, with 57% selecting the most supportive response (i.e., category 4).

**Table 1:** Summary Statistics for Bullying Outcome Variables.

	Full Sample		By Period					
	(2007–2016)		2007–2010		2011–2016		2015–2016	
	Mean	SD	Mean	SD	Mean	SD	Mean	SD
**Bullying Victimization (%)**								
Dummy = 𝕀(Ever been bullied)	20.53	40.39	16.42	37.05	22.38	41.68	20.03	40.03
Categorical variable (%) :								
Category 1	70.47	45.62	83.58	37.05	62.34	48.45	79.97	40.03
Category 2	17.88	38.32	6.01	23.76	23.41	42.34	15.87	36.54
Category 3	4.83	21.45	2.94	16.89	6.32	24.33	2.35	15.16
Category 4	6.81	25.19	7.47	26.30	7.93	27.01	1.81	13.32
**Bullying Perpetration (%)^†^**								
Dummy = 𝕀(Ever bullied others)	8.43	27.79	8.55	27.96	8.37	27.69	-	-
Categorical variable (%):								
Category 1	83.32	37.28	68.72	46.36	91.63	27.69	-	-
Category 2	3.66	18.79	3.58	18.59	3.71	18.90	-	-
Category 3	1.29	11.27	1.37	11.63	1.24	11.05	-	-
Category 4	11.73	32.18	26.32	44.04	3.42	18.19	-	-
**Bystander Intervention (%)**								
Dummy = 𝕀(Willing to intervene)	89.02	31.26	86.81	33.84	89.61	30.52	90.25	29.66
Categorical variable:								
Category 1	5.33	22.46	6.35	24.39	4.97	21.73	5.07	21.93
Category 2	5.65	23.08	6.83	25.23	5.42	22.65	4.68	21.12
Category 3	31.88	46.60	35.16	47.75	31.01	46.25	30.15	45.89
Category 4	57.14	49.49	51.65	49.47	58.61	49.25	60.10	48.97
Observations	3,538,062		871,167		2,083,951		582,944	

*Notes*: Years listed in the column headings refer to Georgia’s school year starting in August of the listed year and ending in May of the next calendar year. For instance, the 2007 school year starts in August 2007 and ends in May 2008. The indicator function 𝕀(·) takes the value 1 if the condition inside the parentheses is true and 0 otherwise. ^†^Surveys corresponding to school years 2015 and 2016 do not contain a bullying perpetration question. Categorical variables: For all bullying outcomes, category 1 corresponds to the absence of bullying involvement (e.g., never bullied), and category 4 represents the highest intensity or frequency of bullying involvement (e.g., bullied every day). Intermediate categories reflect increasing levels of involvement. SD: standard deviation.

A mean difference analysis of outcome variables conditional on school district indicators suggested a 6 percentage point (pp) increase in bullying victimization after the ABL of 2010 (*t* = 23.34, *p*-value ≤ 0.001) and a 3pp decrease after the ABL of 2015 (*t* = 16.40, *p*-value ≤ 0.001). Moreover, while our mean difference analysis did not indicate a significant change in the average number of bullying perpetration reports, it suggested a 3pp increase in the average number of bystanders who were likely to help bullying victims after the passage of the 2010 ABL. These simple mean difference analyses, however, do not account for an array of confounding effects that we take into account in our regression analyses below.

Table 2 presents the descriptive statistics for student-, school-, school district-, and county-level variables used in this study. For instance, we see that 42% of the students in the sample are among racial minorities and on average 54% of students participate in the free and reduced-price school lunch program.

**Table 2:** Summary Statistics for Characteristics of Students, Schools, School Districts, and Counties.

	Mean	SD
**Student characteristics (%)**		
Female	50.79	49.99
Male	49.21	49.99
Middle school	49.76	50.01
High school	50.24	50.01
Non-Hispanic Black	33.92	47.34
Hispanic or Latino	11.31	31.67
White or Caucasian	45.57	49.80
Asian or Pacific Islander	4.17	19.98
Other races	5.03	21.85
Racial minority	42.08	49.37
**Student-level school climate variables (%)**		
Feeling successful at school	86.76	33.89
Feeling safe at school	76.12	42.64
School has clear rules	86.43	34.25
School has high standards	84.43	35.87
**Student-level peer support variables**		
Students get along (%)	88.31	32.13
**Student-level adult support variables**		
Seeking adult help (%)	78.62	41.01
**Student-level risky behavior variables**		
Alcohol use (# of days in last month)	1.07	4.46
Marijuana use (# of days in last month)	0.90	4.46
Adults disapprove of alcohol (%)	84.32	13.22
Adults disapprove of Marijuana (%)	86.75	11.48
**School-level variables**		
FRPL participation rate (%)	54.30	24.48
Pupil service ($ per student)	3.01	0.91
Bullying and intimidation discipline rate (%)	8.11	11.53
Student-reported bullying incidence (%)	20.52	11.26
**School-district-level variables (%)**		
Poverty rate (ages 5–17)	22.43	9.18
School located in rural areas	28.64	45.21
School located in suburban areas	47.87	49.95
School located in city/town	23.48	42.39
**County-level variables**		
Violent crime rate	6.29	8.76
Observations	3,538,062	

*Notes*: FRPL: Free and Reduced-Price Lunch program. SD: standard deviation.

## Empirical methods

We first investigate the relationship between ABLs and the prevalence of bullying behavior among different bullying parties. We begin by estimating the following linear probability model (LPM) separately for bullying victimization and bystander intervention outcomes:

$$Y_{ijdt}^{\left(p\right)}=\beta_{1}+\beta_{2}ABL_{t}^{2010}+\beta_{3}ABL_{t}^{2015}+X_{ijdt}^{\prime }\beta_{4}\;+S_{jdt}^{\prime }\beta_{5}+D_{dt}^{\prime }\beta_{6}+\beta_{7}Crime_{ct}+\delta_{d}+\sum_{r=1}^{2}\omega_{r}Trend_{t}^{r}+\varepsilon_{ijdt}$$ (1)

where $Y_{ijdt}^{\left(p\right)}$ is an indicator variable equal to one if student *i*, attending public school *j* in school district *d*, in school year t∈[2007,2016], reports being a bullying victim (*p* =  victimization) or willingness to intervene as a bystander (*p* =  bystander); *ABL*^2010^ and *ABL*^2015^ are our main policy variables of interest, defined above, and capture the changes in bullying outcomes following the implementation of 2010 and 2015 ABLs, relative to the *status quo* policy (i.e., the 1999 ABL) period. We acknowledge that in the absence of a comparison group to control for other potential contemporaneous policy changes, we may not be able to attribute estimated changes in bullying behavior outcome variables to the causal effects of the ABLs of 2010 and 2015. *X*_*ijdt*_ is a vector of student characteristics listed in Table 2; similarly, $S_{jdt}^{\prime }$ and $D_{dt}^{\prime }$ include a list of control variables at the school and school district levels, respectively, and *Crime*_*ct*_ is the county-level violent crime rate. Other variables in our model include school district fixed effects $\delta_{d}$, to control for time-invariant characteristics of school districts, and a quadratic time trend to capture the impact of unobserved time-variant common factors across all school districts.

As we mentioned earlier, our bullying perpetration outcome variable ($Y_{ijdt}^{\left(\text{perpetration}\right)}$) is constructed for SYs 2007–2013. Thus, we are only able to examine the relationship between the 2010 ABL and the prevalence of bullying perpetration. We do this by estimating a slightly different version of the Eq (1), excluding the 2015 ABL indicator variable ($ABL_{t}^{2015}$).

Despite the ease of interpretation of coefficients from the LPM in Eq (1), one might be concerned about the well-known limitations of this model (e.g., heteroskedasticity, out-of-bound predicted probabilities). To address such concerns while also allowing for nonlinear relationships between our policy variables and bullying outcomes, we estimate the following Probit model:

$$Pr(Y_{ijdt}^{\left(p\right)}=1|Z)=\Phi (\beta_{1}+\beta_{2}ABL_{t}^{2010}+\beta_{3}ABL_{t}^{2015}+X_{ijdt}^{\prime }\beta_{4}\;+S_{jdt}^{\prime }\beta_{5}+D_{dt}^{\prime }\beta_{6}+\beta_{7}Crime_{ct}+\delta_{d}+\sum_{r=1}^{2}\omega_{r}Trend_{t}^{r})$$ (2)

where Pr(·) denotes the conditional probability of observing the outcome variable $Y_{ijdt}^{\left(p\right)}$ taking on a value of one, *Z* is a vector of all right-hand-side variables, and *Φ* is the cumulative distribution function (CDF) of the standard normal distribution. As before, we estimate Eq (2) for p∈{victimization, bystander} and its restricted version, omitting $ABL_{t}^{2015}$, for the bullying perpetration outcome ($Y_{ijdt}^{\left(\text{perpetration}\right)}$).

According to ABLs, for a behavior to be considered bullying, it should be repeated multiple times. Moreover, harsher bullying-related disciplinary actions are taken when bullying is repeated. Therefore, we also test the hypothesis that the effects of ABLs are the same across the entire distribution of bullying behavior (i.e., across different categories) by estimating the following Ordered Probit model:

$$Y_{ijdt}^{\left(p\right)}=k\;\text{if}\;\alpha_{k-1}<Y_{ijdt}^{(p)*}\leq \alpha_{k},\;\;\;Pr(Y_{ijdt}^{\left(p\right)}=k|Z)=\Phi (\alpha_{k}+Z_{ijdt}^{\prime }\beta )-\Phi (\alpha_{k-1}+Z_{ijdt}^{\prime }\beta )$$ (3)

where $Y_{ijdt}^{(p)*}$ is a latent continuous outcome underlying an observed categorical bullying outcome variable $Y_{ijdt}^{\left(p\right)}$ and $\alpha_{k}$ are the thresholds used to define bullying outcome categories k∈{1,2,3,4}, as discussed above.

### Heterogeneity analysis

Next, we examine heterogeneity in the relationship between ABLs and different bullying outcome variables with respect to characteristics of schools, school districts, and counties. To this end, we augment the LPM in Eq (1) with several interaction terms between indicators for ABLs and covariates of interest. We use the LPM specification for this analysis as the interaction effects in a nonlinear model cannot be evaluated simply by looking at the sign, magnitude, or statistical significance of the coefficient [41,42]:

$$Y_{ijdt}^{\left(p\right)}=\beta_{1}+\beta_{2}ABL_{t}^{2010}+\beta_{3}ABL_{t}^{2015}\;+X_{ijdt}^{\prime }\beta_{4}+S_{jdt}^{\prime }\beta_{5}+D_{dt}^{\prime }\beta_{6}+\beta_{7}Crime_{ct}+\delta_{d}+\sum_{r=1}^{2}\omega_{r}Trend_{t}^{r}\;+\left(\sum_{l=1}^{2}\alpha_{1l}ABL_{t}^{2010}\times s_{l,jdt}\right)+\left(\sum_{l=1}^{2}\alpha_{2l}ABL_{t}^{2015}\times s_{l,jdt}\right)\;+\left(\sum_{l=1}^{3}\alpha_{3l}ABL_{t}^{2010}\times d_{l,jdt}\right)+\left(\sum_{l=1}^{3}\alpha_{4l}ABL_{t}^{2015}\times d_{l,jdt}\right)\;+(\alpha_{5}ABL_{t}^{2010}\times Crime_{ct})+(\alpha_{6}ABL_{t}^{2015}\times Crime_{ct})\;+\varepsilon_{ijdt}$$ (4)

where *s*_*l*,*jdt*_ is the *l*^*th*^ school-level characteristic, including pupil service expenditure per student and the percentage of students enrolled in the free and reduced-price school lunch program. Similarly, *d*_*l*,*jdt*_ represents the $l^{\text{th}}$ school district-level characteristic, including the percentage of the population ages 5–17 living in poverty and a categorical indicator for urbanization level (city/town, suburban, or rural), with rural areas serving as the reference group. We also include the county-level violent crime rate *Crime*_*ct*_ and its interactions with the ABL indicators to account for external environmental correlates of bullying.

### School-level analysis of disparity in bullying reporting

We also investigate the existence of disparities in bullying victimization incidents reported by students and school personnel—aggregated at the school level—and whether the reporting disparity, if present, narrowed following the implementation of ABLs. To statistically test for disparities in bullying reporting, we utilize the rank-based (nonparametric) statistical method of Somers’ *D* [43], which measures the ordinal association of random variables [44,45]. For two random variables V and *P*, sampled jointly from a bivariate distribution, consider the following equation:

$$\left\{ \begin{array}{c} \tau_{V,P}=E[\text{sign}(V_{1}-V_{2})\text{sign}(P_{1}-P_{2})]\\ D_{V,P}=\frac{\tau_{V,P}}{\tau_{V,V}} \end{array}\right.$$ (5)

where $\left(V_{1},P_{1}\right)$ and $\left(V_{2},P_{2}\right)$ are bivariate random variables sampled independently from the same population, and *E* denotes expectation. In our context, V denotes the percentage of school-level bullying victimization reported by students, and *P* represents the number of actions taken by school personnel for incidents of threat, intimidation, or bullying, rescaled to a 0–1 range. Somers’ *D* of V with respect to *P*, is defined based on Kendall’s $\tau_{V,P}$ or the difference between the probabilities of concordance and discordance between the reported bullying by victims and school personnel. The V-values and *P*-values are said to be concordant if the larger of the two V-values is associated with the larger of the two *P*-values, and they are said to be discordant if the larger V-value is associated with the smaller *P*-value. A statistically significant Somers’ *D* statistic suggests the presence of disparity in reporting, where a positive $D_{V,P}$ indicates students’ reports stochastically dominate school personnel’s reports of bullying incidents.

Next, we estimate the relationship between ABLs and the school-level bullying victimization reports by students and school personnel using a similar regression framework to our previous analyses:

$$V_{jdt}=\beta_{1}+\beta_{2}ABL_{t}^{2010}+\beta_{3}ABL_{t}^{2015}+\delta_{d}+\sum_{r=1}^{2}\omega_{r}Trend_{t}^{r}+u_{jdt}$$ (6)

$$P_{jdt}=\alpha_{1}+\alpha_{2}ABL_{t}^{2010}+\alpha_{3}ABL_{t}^{2015}+\delta_{d}+\sum_{r=1}^{2}\omega_{r}Trend_{t}^{r}+v_{jdt}$$ (7)

To investigate whether disparities in bullying reporting became narrower following the implementation of ABLs, we estimate the Somers’ *D* for the residuals of Eqs (6) and ((7)).

## Results

Table 3 presents results based on Eq (1), estimated separately for our three bullying outcomes: victimization, perpetration, and bystander intervention. Results in the first column show significant declines in the prevalence of reported bullying victimization following the implementation of the 2010 and 2015 ABLs. Notably, and consistent with intuition, we observe a larger reduction of about 22pp in reported incidents when the more stringent 2015 ABL went into effect (9pp vs. 31pp reductions). Estimates in the second column indicate a slight increase of about 2pp in the likelihood of reported bullying perpetration following the implementation of the 2010 ABL. One possible explanation is based on the psychological reactance theory, which suggests that prevention programs such as ABLs that restrict behavioral freedoms may elicit emotional responses, negative cognition, and efforts to reassert decision-making control [46–48]. Therefore, a slight increase in the prevalence of reported bullying perpetration following the implementation of an ABL is conceptually plausible. Finally, the third column presents the impact of ABLs on students who report a high willingness to intervene as bystanders during bullying incidents. Following the enactment of the 2010 ABL, students were more likely to indicate a willingness to intervene to help those being bullied, but no such effect is observed for the 2015 ABL, as indicated by its statistically insignificant coefficient estimate.

**Table 3:** Relationship between Anti-Bullying Laws (ABLs) and Bullying Behavior of Different Parties: Linear Probability Model.

	(1) Bullying Victimization	(2) Bullying Perpetration	(3) Bystander Intervention
*ABL* ^2010^	−8.97^***^	1.70^***^	1.70^***^
	(0.39)	(0.34)	(0.17)
*ABL* ^2015^	−30.89^***^		−0.25
	(0.95)		(0.17)
Male	−1.64^***^	−0.04	−4.42^***^
	(0.18)	(0.12)	(0.12)
*Student’s School Level (Reference: Middle school)*			
High school	−9.32^***^	−3.35^***^	−0.86^***^
	(0.33)	(0.14)	(0.10)
*Student’s Race/Ethnicity (Reference: Non-Hispanic Black*)			
Hispanic or Latino	−0.68^**^	−2.49^***^	1.44^***^
	(0.26)	(0.13)	(0.34)
White or Caucasian	5.35^***^	−2.15^***^	4.08^***^
	(0.19)	(0.11)	(0.21)
Asian or Pacific Islander	2.55^***^	−1.73^***^	0.05
	(0.49)	(0.30)	(0.35)
Other races	3.99^***^	−0.08	2.29^***^
	(0.34)	(0.14)	(0.18)
Racial minority	0.81^***^	0.40^***^	0.23
	(0.17)	(0.11)	(0.20)
Feeling successful at school	−6.81^***^	−4.15^***^	1.88^***^
	(0.14)	(0.12)	(0.12)
Feeling safe at school	−11.72^***^	−3.80^***^	4.13^***^
	(0.35)	(0.13)	(0.07)
School has clear rules	−3.15^***^	−3.35^***^	7.21^***^
	(0.17)	(0.12)	(0.19)
School has high standards	−1.21^***^	−2.05^***^	6.39^***^
	(0.10)	(0.07)	(0.27)
Students get along	−11.77^***^	−6.96^***^	14.86^***^
	(0.30)	(0.18)	(0.33)
Seeking adult help	0.39^***^	−0.80^***^	8.64^***^
	(0.15)	(0.06)	(0.24)
Alcohol use		0.50^***^	−0.07^***^
		(0.02)	(0.01)
Marijuana use		0.51^***^	−0.10^***^
		(0.02)	(0.01)
Adults disapprove alcohol		−1.95^***^	3.83^***^
		(0.09)	(0.16)
Adults disapprove marijuana		−1.90^***^	5.65^***^
		(0.11)	(0.12)
*Reference: Rural areas*			
Suburban areas	−0.68^***^	−0.27	0.09
	(0.26)	(0.17)	(0.12)
City/Town	−0.12	−0.24	0.18
	(0.34)	(0.18)	(0.16)
FRPL participation rate	1.13^*^	0.45	−0.81^*^
	(0.67)	(0.38)	(0.44)
Pupil service ($ per student)	1.54^*^	−0.79^**^	−0.08
	(0.87)	(0.31)	(0.13)
Poverty rate (ages 5–17)	8.84	6.83	3.45
	(8.94)	(7.23)	(2.90)
Violent crime rate	0.23	0.16	−0.08^**^
	(0.18)	(0.10)	(0.03)
Constant	53.36^***^	37.76^***^	40.65^***^
	(3.36)	(1.92)	(0.88)
School district FEs	Yes	Yes	Yes
Quadratic trend	Yes	Yes	Yes
Observations	3,538,062	2,401,169	3,538,061

*Notes*: FRPL: Free and Reduced-Price Lunch program. Coefficient estimates are multiplied by 100 for ease of interpretation. Standard errors in parentheses are clustered at the school district level. * *p*<0.10, ** *p*<0.05,eak *** *p*<0.01.

Coefficient estimates for covariates in Table 3 also provide useful insights into reported bullying outcomes. First, results suggest that male students are less likely to report bullying victimization compared to female students (column 1), equally likely to report bullying perpetration (column 2), and less likely to intervene as bystanders (column 3). Further, estimates across all three columns indicate that high school students are, on average, less likely to be involved in bullying incidents—either as victims, perpetrators, or bystanders. This finding is consistent with previous studies that investigate the prevalence of bullying between middle and high school students [49]. Among racial/ethnic groups in the survey, Hispanic/Latino students are less likely to report bullying victimization than Non-Hispanic Black students (the reference group), whereas Non-Hispanic White/Caucasian students, Asian and Pacific Islander students, and those from other racial groups are more likely to report being bullied. Our models also control for being in a racial minority (variable “Racial minority") and examine its association with reported bullying outcomes. We find that students identified as part of a racial minority group are more likely to report both bullying victimization and perpetration.

As found in previous studies [50], school climate plays an important role in reducing the number of bullying incidents. All four positive school climate indicators in our models—feeling successful, feeling safe at school, school has clear rules, school has high standards—are negatively associated with reports of both bullying victimization and bullying perpetration with estimated effects ranging from -1.2pp to -11.8pp. Among these, the indicator of feeling safe at school shows the strongest association. A positive school climate is also associated with a greater likelihood of bystander intervention, with estimated effects ranging from 1.9pp to 7.2pp. Students who feel successful are less likely to report being victimized or engaging in perpetration, and more likely to intervene as bystanders against bullying behavior.

Moving down in Table 3, the estimated coefficient for the variable “Students get along” is statistically significant and negative for both bullying victimization and perpetration outcomes. This result is consistent with previous studies showing that students who engage in bullying perpetration may also be victims of bullying—the so-called “bully-victims”—due to their resemblance to victims in being rejected and isolated by their peers [51]. Seeking adult help is associated with a higher probability of bullying victimization by 0.39pp. This relatively weak association suggests that some victims may be uncertain about whether their experience is serious enough to report, underscoring the importance of encouraging students to talk to an adult about their experience. Estimated coefficients for other variables can be interpreted similarly and generally conform to intuition. For example, alcohol and marijuana use are associated with a higher probability of bullying perpetration, whereas adult disapproval of alcohol and marijuana use is associated with a lower likelihood.

Estimated marginal effects from the Probit model in Panel (A) of Table 4 for all bullying outcomes are qualitatively consistent with those from the LPM in Eq (1). In particular, column (1) shows that the implementation of the 2010 ABL was associated with a 9pp reduction in the probability of bullying victimization, whereas the 2015 ABL was associated with a larger decline of about 20pp. Coefficient estimates for the Probit model are presented in the Appendix Table A1 (S1 Text).

**Table 4:** Relationship between Anti-Bullying Laws (ABLs) and Bullying Behavior of Different Parties: Probit and Ordered Probit Models.

			(1) Bullying Victimization	(2) Bullying Perpetration^†^	(3) Bystander Intervention
**(A) Probit**	*ABL* ^2010^		−9.134^***^	1.592^***^	1.561^***^
			(0.405)	(0.322)	(0.167)
	*ABL* ^2015^		−19.92^***^		−0.379^**^
			(0.422)		(0.153)
**(B) Ordered Probit**	*ABL* ^2010^	Category 1	16.98^***^	26.65^***^	−0.789^***^
			(0.757)	(2.098)	(0.0749)
		Category 2	−6.483^***^	−4.111^***^	−0.626^***^
			(0.245)	(0.234)	(0.0547)
		Category 3	−2.530^***^	−1.686^***^	−1.548^***^
			(0.0942)	(0.106)	(0.134)
		Category 4	−7.970^***^	−20.86^***^	2.963^***^
			(0.473)	(1.822)	(0.260)
	*ABL* ^2015^	Category 1	36.38^***^		0.279^***^
			(0.334)		(0.0977)
		Category 2	−15.48^***^		0.222^***^
			(0.305)		(0.0757)
		Category 3	−4.970^***^		0.557^***^
			(0.0535)		(0.183)
		Category 4	−15.94^***^		−1.058^***^
			(0.140)		(0.356)

*Notes*: Marginal effects from Probit and Ordered Probit models are reported and are multiplied by 100 for ease of interpretation. Categorical variables: For all bullying outcomes, category 1 corresponds to the absence of bullying involvement (e.g., never bullied or not willing to intervene), while category 4 represents the highest intensity or frequency of bullying involvement (e.g., bullied every day or very willing to intervene). Intermediate categories reflect increasing levels of involvement. ^†^Surveys corresponding to school years 2015 and 2016 do not include a bullying perpetration question; thus, *ABL*^2015^ is not analyzed for perpetrators. Standard errors in parentheses are clustered at the school district level. * *p*<0.10, ** *p*<0.05, *** *p*<0.01.

The estimated marginal effects from the Ordered Probit model are presented in Panel (B) of Table 4, and coefficient estimates are reported in Appendix Table A2 (S1 Text). These results provide additional support for our findings discussed above. First, in column (1), we observe an increase in the proportion of students reporting never experiencing bullying victimization (category 1) and a decrease in the fraction of students reporting victimization at varying intensities (categories 2–4) following the implementation of the 2010 and 2015 ABLs, with a larger estimated association for the 2015 ABL. A similar pattern is observed in column (2) for bullying perpetration following the 2010 ABL, which is associated with a 21pp reduction in the likelihood of repeated perpetration among the 12% of students who reported bullying others multiple times in the past 30 days (category 4). Moreover, in column (3), we see a reduction in the share of students who would be less willing or not willing (i.e., categories 1–3) to engage in bullying incidents to help those being bullied and an increase in the fraction of students who would be highly willing to get involved in the incidents to help the victims. On the other hand, the more stringent ABL of 2015 was associated with a slight reduction in the share of students who would be highly willing to get involved in bullying incidents to help the victims (category 4).

### Heterogeneity analysis results

Table 5 presents estimation results from our heterogeneity analysis with respect to external environment factors for bullying victimization and bystander intervention outcomes. The heterogeneous impact of ABLs may differ depending on the level at which the analysis is conducted (i.e., school district vs. county level). Although school-district-level policies are more likely to impact (or confound) anti-bullying policies than county-level policies, we implemented a statistical test of equality of the estimated coefficients of ABLs from models that controlled for school-district fixed effects and those that conditioned on county fixed effects. We did not find statistically significant differences in the impact of ABLs between the within-school-district analysis compared to the within-county analysis. As such, we report results from the models that controlled for school-district fixed effects.

**Table 5:** Heterogeneity in the Relationship between Anti-Bullying Laws (ABLs) and Bullying Behavior by External Environment Characteristics, Linear Probability Model.

	(1) Bullying Victimization	(2) Bystander Intervention
*ABL* ^2010^	−7.339^***^	7.561^***^
	(1.506)	(1.423)
*ABL* ^2015^	−30.87^***^	−0.00160
	(1.179)	(1.530)
Pupil service ($ per student)	1.374^*^	−0.243
	(0.823)	(0.475)
*ABL*^2010^ × Pupil service ($ per student)	0.452	−0.304
	(0.497)	(0.391)
*ABL*^2015^ × Pupil service ($ per student)	−0.464	0.0696
	(0.360)	(0.338)
FRPL participation rate (%)	1.155	−3.916^***^
	(0.771)	(1.465)
*ABL*^2010^ × FRPL participation rate (%)	−0.949	0.781
	(1.035)	(1.431)
*ABL*^2015^ × FRPL participation rate (%)	3.418^***^	−6.672^***^
	(0.594)	(1.429)
Poverty rate (ages 5–17) (%)	19.36^*^	11.29
	(10.38)	(9.876)
*ABL*^2010^ × Poverty rate (ages 5–17) (%)	−10.66**	−7.560*
	(4.342)	(4.463)
*ABL*^2015^ × Poverty rate (ages 5–17) (%)	1.829	15.66^***^
	(2.798)	(4.387)
Suburban areas (Reference: Rural areas)	0.340	−0.313
	(0.483)	(0.755)
City/Town (Reference: Rural areas)	−0.545	−0.169
	(0.572)	(0.729)
*ABL*^2010^ × Suburban areas	−1.219**	0.824
	(0.504)	(0.766)
*ABL*^2010^ × City/Town	0.670	0.755
	(0.560)	(0.746)
*ABL*^2015^ × Suburban areas	−0.556	−0.818
	(0.385)	(0.645)
*ABL*^2015^ × City/Town	−0.280	−0.986
	(0.438)	(0.790)
Violent crime rate	0.233	−0.217^*^
	(0.198)	(0.128)
*ABL*^2010^ × Violent crime rate	0.106	−0.0659**
	(0.0717)	(0.0314)
*ABL*^2015^ × Violent crime rate	−0.0839	−0.0490*
	(0.0509)	(0.0279)
Constant	52.16^***^	217.3^***^
	(3.282)	(3.134)
Other individual level control variables	Yes	Yes
School district FEs	Yes	Yes
Quadratic trend	Yes	Yes
Observations	3,538,062	3,538,054

*Notes*: Coefficient estimates are multiplied by 100. Standard errors in parentheses are clustered at the school district level. * *p*<0.10, ** *p*<0.05, *** *p*<0.01.

In column (1), we see a positive association between pupil service expenditure per student and self-reported bullying victimization, with no significant change in this association following the passage of the two ABLs. However, the implementation of the 2015 ABL is associated with an increase of about 3.4pp in the self-reported victimization in schools with a large percentage of participants in the free and reduced lunch programs. Furthermore, while living in a neighborhood with a higher poverty rate among children aged 5–17 is correlated with a higher probability of experiencing bullying victimization (by about 19.4pp), this correlation is substantially weaker (by about 10.7pp) following the implementation of the 2010 ABL. Moreover, while we fail to reject the null hypothesis that the probability of bullying victimization differs between towns/cities and rural areas prior to the ABLs, we observe a slight reduction in suburban areas after the implementation of the 2010 ABL.

The results for bystander intervention in column (2) suggest that a higher participation rate in the free and reduced-price school lunch programs is associated with a reduction in the share of students who would be willing to intervene in bullying incidents following the 2015 ABL. The results regarding poverty rates are mixed: while the 2010 ABL is associated with a decrease in the likelihood of bystander intervention, the 2015 ABL is associated with an increase. Additionally, in counties with higher violent crime rates, students are less likely (by 0.22pp) to express their willingness to intervene to help bullying victims, and this negative association becomes stronger after both the 2010 and 2015 ABLs.

### Disparity in bullying reporting results

In Table 6, Panel (A), we investigate the relationship between ABLs and school-level reports of bullying victimization from the perspectives of students and school personnel. In column (1), we find results consistent with the student-level analysis reported above: both ABLs were negatively associated with student-reported victimization, with the 2015 ABL linked to a larger decline of about 20pp than the 2010 ABL. In the second column, however, we find no significant change in bullying victimization reported by school personnel following the implementation of 2015 ABL.

**Table 6:** Relationship between School-Level Reports of Bullying Victimization and Anti-Bullying Laws (ABLs).

	(1) Bullying Reported by Students	(2) Bullying Reported by School Personnel
**Panel (A)**		
*ABL* ^2010^	−8.331^***^	1.732^**^
	(0.236)	(0.639)
*ABL* ^2015^	−28.68^***^	−0.0517
	(0.644)	(0.345)
Constant	17.08^***^	3.956^***^
	(0.264)	(0.699)
School district FEs	Yes	Yes
Quadratic trend	Yes	Yes
Observations	8,618	8,504
**Panel (B)**		
Estimated Somers’ D statistics:		
Students vs. school personnel (outcome variables)		0.432^***^
		(0.003)
Students vs. school personnel (regression residuals)		0.007
		(0.005)

*Notes*: Coefficient estimates in Panel A are multiplied by 100. Standard errors in parentheses in Panel A are clustered at the school district level. Jackknife standard errors in parentheses for Somers’ D test statistics in Panel B. * *p*<0.05, ** *p*<0.01, *** *p*<0.001.

In Panel (B), we examine reporting discrepancies between students and school personnel using Somers’ D statistics. The first row of Panel (B) shows that students are 43% more likely to report having been bullied compared to what is recorded by school officials, indicating a substantial gap in perceptions or documentation of bullying incidents across reporting sources. Several possible explanations for the original divergence between the two distributions include students’ fear of retaliation when school officials are involved in bullying incidents, or potential overreporting of victimization in student self-reports. Importantly, the second row shows that this reporting discrepancy disappears once we control for the implementation of the 2010 and 2015 ABLs, suggesting that these laws have helped align student and school personnel reporting. The corresponding cumulative distribution functions for bullying victimization reported by students and school officials are shown in the upper panel of Appendix Fig A1 (S1 Text), illustrating that school reports stochastically dominate student reports in the first order. The lower panel presents the adjusted distributions after accounting for ABLs, which is consistent with the narrowing of discrepancies indicated by the residual-based Somers’ D statistic.

## Conclusion and discussion

In this paper, we utilize unique administrative data collected by the GaDOE from all public schools in Georgia, for students in grades 6–12 over SYs 2007–2016, to explore the relationship between the state’s ABLs and changes in bullying behavior of different bullying parties (victims, perpetrators, bystanders). In particular, we examine how these relationships might vary by the stringency level of the ABLs and external environment characteristics, among other factors. We also analyze disparities in bullying reporting by students and school personnel at the school level.

Our results suggest that Georgia’s ABLs of 2010 and 2015—with the latter being more stringent due to the addition of cyberbullying laws—were associated with significant reductions in the prevalence of students’ self-reports of bullying victimization. We find the more stringent ABL of 2015 was associated with substantially larger declines (about 10-20pp, depending on the model used) in reports of bullying victimization compared to the less stringent ABL of 2010. Importantly, we find this association to be stronger for the more at-risk students, who reported being bullied multiple times during the past 30 days.

Regarding bullying perpetration, results from our Ordered Probit model indicate that after the implementation of the 2010 ABL, students who reported bullying others on a daily basis were less likely to continue doing so. Our findings from the analysis of bystander intervention suggest that students were slightly more likely to intervene in bullying incidents to help victims if they witnessed such incidents. This is an important finding from a public health perspective, given the important role of bystanders in reducing bullying behavior. Lynn Hawkins, Pepler, and Craig (2001) [52] found that more than half of bullying situations stop when a peer intervenes on behalf of the student being bullied. This positive association, however, is not evident for the 2015 ABL. One possible explanation is that the objective of the 2015 ABL was primarily to clarify the definition of cyberbullying and define appropriate disciplinary actions. Therefore, the stricter enforcement of more stringent laws might have convinced bystanders that their intervention was not necessary. An alternative explanation could be that the fear of bullies receiving harsher punishments, if exposed, might have discouraged bystanders from intervening.

In our analyses, we also investigated the influence of being in a racial/ethnic minority group on the incidence of bullying victimization. We find that being a racial/ethnic minority—relative to the school’s racial/ethnic composition—is positively associated with the likelihood of experiencing bullying victimization. This finding is in contrast with studies reporting no association or mixed evidence of association between racial/ethnic differences and bullying victimization [53,54], but aligns with a body of literature that documents minority racial/ethnic status as a risk-factor that increases the likelihood of being bullied [39,55–58]. We also note that the ethnic mix of the school plays a critical role in mitigating this risk; as shown by [58], greater diversity can reduce the negative influence of minority status to an insignificant level. This highlights the importance of further research into how school demographic composition shapes bullying dynamics.

Our findings from the heterogeneity analysis based on external environment characteristics further highlight the importance of the passage of localized anti-bullying policies. For instance, with regard to participation in the free and reduced-price lunch program, we find that the implementation of the more stringent 2015 ABL is associated with an increase in the prevalence of reported bullying victimization in schools with a larger share of students participating in the subsidized school lunch program. Furthermore, while we fail to reject the null hypothesis that the probability of bullying victimization differs in towns and cities compared to rural areas prior to the two ABLs, we observe a slight reduction in suburban areas following the implementation of the 2010 ABL. Moreover, we find that while the 2010 ABL is estimated to be more effective at reducing self-reported bullying victimization and less effective in encouraging bystander intervention in areas with higher poverty rates among children aged 5–17, the more stringent 2015 ABL is associated with a larger increase in bystanders’ willingness to intervene.

Lastly, in our analysis of disparities in bullying victimization reporting by students and school personnel, we find that students are more likely to self-report bullying victimization and are also more responsive to the passage of more stringent ABLs. This finding underscores the importance of considering multiple perspectives in bullying incidents and provides valuable insight into the accuracy of bullying reports—information that may help address disputes between parents and school personnel regarding bullying.

Taken together, our findings point to several actionable recommendations for policymakers and school administrators. First, the greater effectiveness of the 2015 ABL in reducing bullying victimization, particularly among students repeatedly targeted, highlights the value of strengthening anti-bullying legislation to address evolving forms of bullying such as cyberbullying. Policymakers should consider periodic updates to anti-bullying laws that reflect changes in communication technologies and social media use among youth. In addition, our findings on the variation in effectiveness across socioeconomic contexts suggest that complementary, locally tailored interventions, such as targeted support in schools with high poverty rates or large shares of subsidized lunch participants, may be necessary to enhance the reach and equity of statewide policies.

For school administrators, the findings underscore the importance of fostering a school climate that supports student reporting and peer intervention. Given that student self-reports respond more strongly to policy changes than school personnel reports, schools should invest in systems that elevate student voices and provide safe, anonymous ways to report incidents. Moreover, the mixed results on bystander intervention call for additional training and resources to empower students to intervene effectively and safely when witnessing bullying. Programs that clarify when peer intervention is encouraged versus when adults should be notified may help address concerns stemming from stricter disciplinary environments. Administrators should also engage in culturally responsive strategies to address bullying of racial/ethnic minorities, especially in schools with limited diversity, and actively monitor the alignment between the school’s demographic composition and the inclusivity of its anti-bullying initiatives.

## Supporting information

S1 Text Appendix. (PDF)
