# Supplementary material for: Heterogeneous impact of anti-bullying laws on school bullying in the State of Georgia
Source: PLoS One. 2025 Sep 26;20(9):e0332028. doi: 10.1371/journal.pone.0332028 (PMC12469156; doi:10.1371/journal.pone.0332028)
Supplement: S1 Text — (PDF) [file pone.0332028.s001.pdf]

Heterogeneous Impact of Anti-Bullying Laws on School Bullying in the State of Georgia

Mona Ahmadiani[1*], Pourya Valizadeh[1], Genti Kostandini[2], Jeffrey L. Jordan[2]

1 Department of Agricultural Economics, Texas A&M University, College Station, Texas, United States
2 Department of Agricultural and Applied Economics, University of Georgia, Athens, Georgia, United States

* Corresponding author
mona.ahmadiani@ag.tamu.edu

Appendices

Table A1: Relationship between Anti-Bullying Laws (ABLs) and Bullying Behavior of Different Parties: Probit Model's Estimated Coefficients

	(1)<br>Bullying<br>Victimization	(2)<br>Bullying<br>Perpetration	(3)<br>Bystander<br>Intervention
ABL^{2010}	-0.34***	0.12***	0.10***
	(0.01)	(0.02)	(0.01)
ABL^{2015}	-1.06***		-0.02**
	(0.03)		(0.01)
Male	-0.06***	0.00	-0.28***
	(0.01)	(0.01)	(0.01)
Student's School Level (Reference: Middle school)			
High school	-0.37***	-0.22***	-0.08***
	(0.01)	(0.01)	(0.01)
Student's Race/Ethnicity (Reference: Non-Hispanic Black)			
Hispanic or Latino	-0.03***	-0.17***	0.08***
	(0.01)	(0.01)	(0.02)
White or Caucasian	0.20***	-0.15***	0.27***
	(0.01)	(0.01)	(0.01)
Asian or Pacific Islander	0.10***	-0.14***	0.01
	(0.02)	(0.02)	(0.02)
Other Race	0.15***	-0.00	0.13***
	(0.01)	(0.01)	(0.01)
Racial minority	0.03***	0.02***	0.01
	(0.01)	(0.01)	(0.01)
Feeling successful at school	-0.23***	-0.22***	0.09***
	(0.00)	(0.00)	(0.01)
Feeling safe at school	-0.42***	-0.24***	0.22***
	(0.01)	(0.01)	(0.00)
School has clear rule	-0.11***	-0.17***	0.29***
	(0.01)	(0.01)	(0.01)

School has high standard	-0.05***	-0.11***	0.27***
	(0.00)	(0.00)	(0.01)
Student get along	-0.37***	-0.32***	0.58***
	(0.01)	(0.01)	(0.01)
Seeking adults help	0.00	-0.06***	0.41***
	(0.01)	(0.00)	(0.01)
Alcohol use		0.02***	-0.00***
		(0.00)	(0.00)
Marijuana use		0.02***	-0.00***
		(0.00)	(0.00)
Adult disapprove alcohol		-0.13***	0.19***
		(0.01)	(0.01)
Adults disapprove marijuana		-0.09***	0.22***
		(0.01)	(0.00)
Reference: Rural areas			
Suburban areas	-0.03***	-0.02*	0.01
	(0.01)	(0.01)	(0.01)
City/Town	-0.01	-0.02	0.01
	(0.01)	(0.01)	(0.01)
FRPL participation rate	0.05*	0.08***	-0.11***
	(0.03)	(0.03)	(0.02)
Pupil service ($ per student)	0.05*	-0.06***	0.01
	(0.03)	(0.02)	(0.01)
Poverty rate (ages 5–17)	0.16	0.46	0.07
	(0.31)	(0.48)	(0.18)
Violent crime rate	0.01	0.01	-0.00**
	(0.01)	(0.01)	(0.00)
School district FEs	Yes	Yes	Yes
Quadratic trend	Yes	Yes	Yes
Constant	0.29**	0.24**	-0.71***
	(0.11)	(0.12)	(0.04)
Observations	3,538,062	2,401,001	3,538,061

Notes: FRPL: Free and Reduced-Price Lunch program. Standard errors in parentheses are clustered at the school district level.

* $p < 0.10$, ** $p < 0.05$, *** $p < 0.01$

Table A2: Relationship between Anti-Bullying Laws (ABLs) and Bullying Behavior of Different Parties: Ordered Probit Model's Estimated Coefficients

	(1) Bullying Victimization	(2) Bullying Perpetration	(3) Bystander Intervention
ABL^{2010}	-0.60***	-1.12***	0.08***
	(0.03)	(0.08)	(0.01)
ABL^{2015}	-2.31***		-0.03***
	(0.04)		(0.01)
Male	-0.05***	-0.00	-0.21***
	(0.01)	(0.01)	(0.00)
Student's School Level (Reference: Middle school)			
High school	-0.33***	-0.23***	-0.22***
	(0.01)	(0.02)	(0.01)
Student's Race/Ethnicity (Reference: Non-Hispanic Black)			
Hispanic or Latino	-0.05***	-0.13***	0.05***
	(0.01)	(0.01)	(0.01)
White or Caucasian	0.14***	-0.10***	0.16***
	(0.01)	(0.01)	(0.01)
Asian or Pacific Islander	0.05***	-0.09***	-0.09***
	(0.02)	(0.02)	(0.01)
Other Races	0.13***	-0.00	0.11***
	(0.01)	(0.01)	(0.01)
Racial minority	0.02***	0.01	0.01
	(0.01)	(0.01)	(0.01)
Feeling successful at school	-0.20***	-0.16***	0.05***
	(0.00)	(0.00)	(0.00)
Feeling safe at school	-0.39***	-0.16***	0.12***
	(0.01)	(0.01)	(0.00)
School has clear rule	-0.11***	-0.12***	0.22***
	(0.00)	(0.01)	(0.01)
School has high standard	-0.04***	-0.07***	0.21***
	(0.00)	(0.01)	(0.01)
Student get along	-0.35***	-0.25***	0.46***
	(0.01)	(0.01)	(0.01)
Seeking adults help	0.01	-0.04***	0.36***
	(0.01)	(0.00)	(0.01)
Alcohol use		0.02***	-0.00***
		(0.00)	(0.00)
Marijuana use		0.02***	-0.00***
		(0.00)	(0.00)
Adults disapprove alcohol		-0.10***	0.16***
		(0.01)	(0.00)
Adults disapprove marijuana		-0.05***	0.15***
		(0.00)	(0.00)
Reference: Rural areas			
Suburban areas	-0.02**	-0.03	0.00
	(0.01)	(0.02)	(0.01)
City/Town	-0.01	-0.05**	0.00
	(0.01)	(0.02)	(0.01)
FRPL participation rate	0.02	0.17***	-0.09***
	(0.02)	(0.06)	(0.01)

Pupil service ($ per student)	0.09*	0.17	0.00
	(0.05)	(0.12)	(0.01)
Poverty rate (ages 5–17)	0.29	-0.46	0.03
	(0.61)	(2.02)	(0.13)
Violent crime rate	0.02	-0.05	-0.00**
	(0.01)	(0.03)	(0.00)
School district FEs	Yes	Yes	Yes
Quadratic trend	Yes	Yes	Yes
μ_1 Constant	-0.67***	1.62***	-0.28***
	(0.19)	(0.52)	(0.05)
μ_2 Constant	0.15	1.81***	0.18***
	(0.20)	(0.52)	(0.05)
μ_3 Constant	0.46**	1.89***	1.34***
	(0.20)	(0.52)	(0.04)
Observations	3,538,054	2,401,169	3,538,054

Notes: FRPL: Free and Reduced-Price Lunch program. Standard errors in parentheses are clustered at the school district level.

* $p < 0.10$, ** $p < 0.05$, *** $p < 0.01$

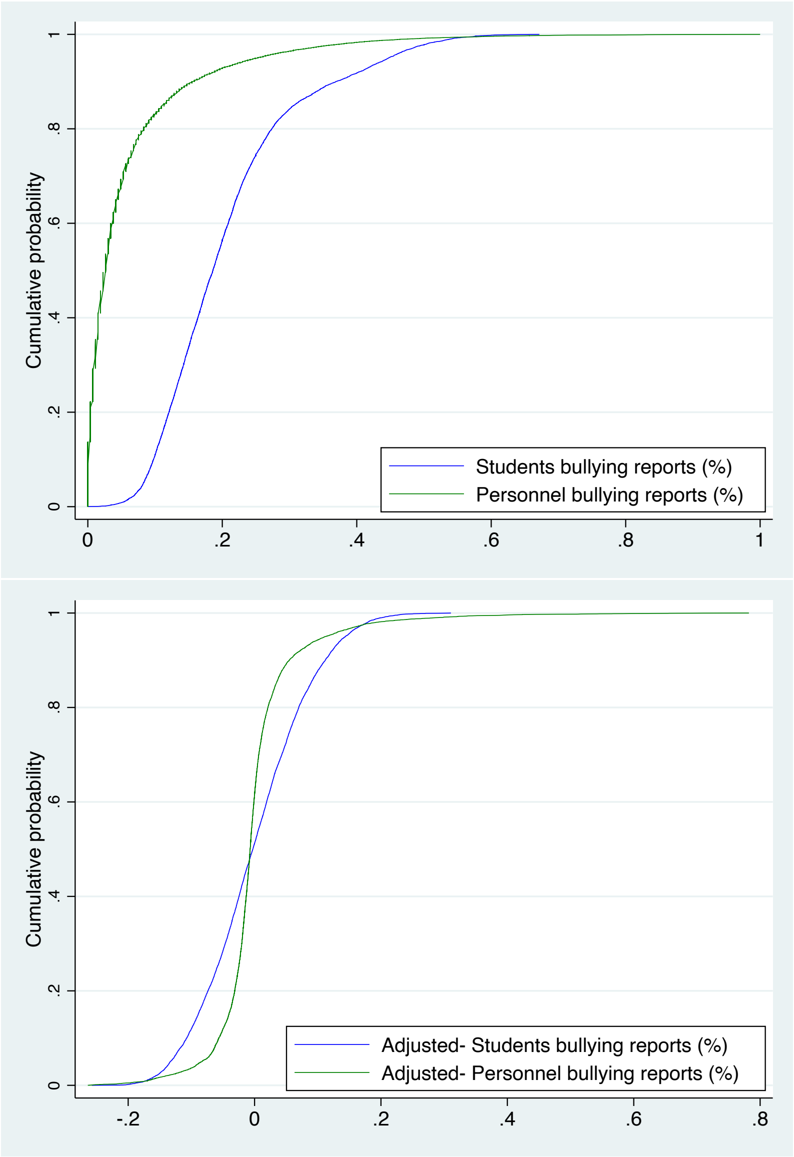

Figure A1: Cumulative Probability Functions (CDF) for Bullying Reporting
Notes: The top panel shows the CDFs for school-level reports of bullying victimization by students and school personnel. The bottom panel plots the CDFs for residuals of regression models in equations (6) and (7), analyzing the link between anti-bullying laws of 2010 and 2015 and school-level reports of bullying by students and school personnel.
